# Quantitative phenotypic screens of *Aspergillus niger* mutants in solid and liquid culture

**DOI:** 10.1016/j.xpro.2022.101883

**Published:** 2022-11-24

**Authors:** Timothy Cairns, Xiaomei Zheng, Claudia Feurstein, Ping Zheng, Jibin Sun, Vera Meyer

**Affiliations:** 1 Chair of Applied and Molecular Microbiology, Institute of Biotechnology, Technische Universität Berlin, 10263 Berlin, Germany; 2 Tianjin Institute of Industrial Biotechnology, Chinese Academy of Sciences, Tianjin 300308, China; 3 Key Laboratory of Systems Microbial Biotechnology, Chinese Academy of Sciences, Tianjin 300308, China; 4 University of Chinese Academy of Sciences, Beijing 100049, China; 5 National Technology Innovation Center of Synthetic Biology, Tianjin 300308, China

**Keywords:** Genetics, Microbiology, Model Organisms, Molecular Biology, Biotechnology and bioengineering

## Abstract

This protocol describes procedures for quantifying *Aspergillus niger* growth in both solid and liquid culture. Firstly, by comparing radial growth between mutant and progenitor isolates on solid agar supplemented with sublethal stressors, susceptibility coefficients can be calculated. Secondly, analysis of macromorphological growth types in liquid culture allows full quantification of how a gene of interest affects submerged growth. By combining these assays, an extensive and quantitative dataset of how a gene of interest impacts growth in this fungus is possible.

For complete details on the use and execution of this protocol, please refer to Cairns et al. (2019)^1^ and Cairns et al. (2022).^2^

## Highlights

• Inoculate *Aspergillus niger* in simple solid and liquid growth media
• Generate quantitative measurements of strain fitness on sublethal stress
• Analyze macromorphology during submerged culture
• Obtain a detailed and quantitative picture of strain growth

## Abstract (editor highlights)

Publisher’s note: Undertaking any experimental protocol requires adherence to local institutional guidelines for laboratory safety and ethics.

## Graphical abstract

**Figure:**
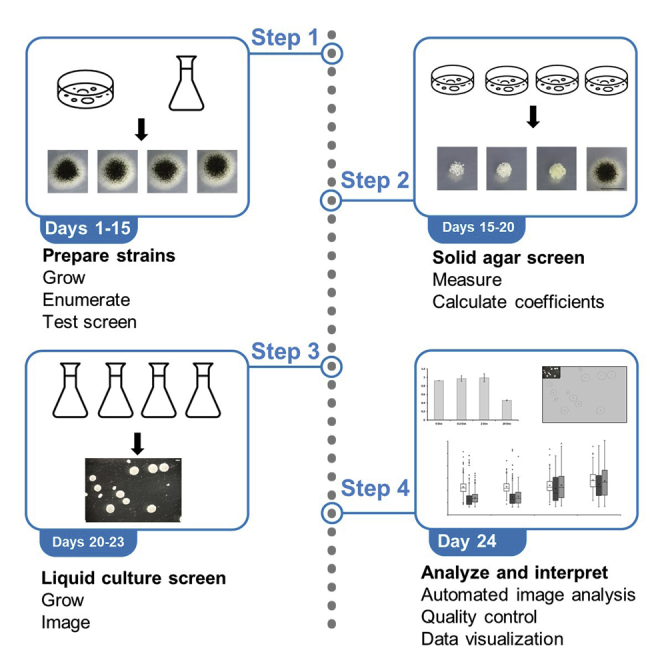

## Before you begin

This protocol details quantification of growth using tet-on conditional expression mutants in *A. niger* isolate MA70.15.^3^ However, this protocol can be adapted for any progenitor and derivative isolate(s), including classical gene deletion/disruption, constitutive overexpression, or mutagenized strains. Moreover, while we exclusively refer to *A. niger*, this protocol can be adapted to any filamentous fungus which is able to grow under standard laboratory culture. For the solid agar experiments, we describe several stress conditions. However, this list is not exhaustive, and many others can be added to enable further predictions of gene function. Finally, we describe applications of the morphology of pelleted and dispersed (MPD) image analysis in a single growth media (minimal media, MM). However, it is applicable to many different liquid media and cultivation conditions.

### Institutional permissions

This protocol uses the filamentous ascomycete fungus *A. niger*, which is has generally regarded as safe (GRAS) status. Nevertheless, all safety certification for use of sporulating fungi should be obtained from relevant authorities and followed carefully. Additionally, this protocol involves the analysis of genetically modified strains, and therefore necessary permission to work with these organisms should be obtained prior to commencing this protocol. All work with *A. niger* described in this protocol should be conducted in a class II microbiological safety cabinet.

### Download and install ImageJ/Fiji and the MPD plugin

Analysis of liquid growth is conducted using ImageJ/Fiji.^4^ Download the current version from: https://imagej.net/software/fiji/downloads. The updated version of the MPD plugin^1^ is contained in Data S1. To install, close ImageJ/Fiji, unzip the Data S1, and copy it to the ‘plugins’ folder contained in ImageJ/Fiji directory.

## Key resources table

**Table:** 

REAGENT or RESOURCE	SOURCE	IDENTIFIER
**Experimental models: Organisms/strains**		

*A. niger* MA70.15	(Meyer et al.^3^)	Not applicable
*A. niger* TC18.1	(Cairns et al.^1^)	Not applicable
*A. niger* TC18.3	(Cairns et al.^1^)	Not applicable

**Chemicals, peptides, and recombinant proteins**		

Agar, granulated	Formedium	Cat#AGR10
CaCl_2_	Sigma-Aldrich	Cat#C1016
Caspofungin diacetate	Sigma-Aldrich	Cat#SML0425
CoCl_2_·6H_2_O	Sigma-Aldrich	Cat#C8661
Congo red	Sigma-Aldrich	Cat#C6767
CuSO_4_·5H_2_O	Sigma-Aldrich	Cat#C8027
Doxycycline hydrochloride	Sigma-Aldrich	Cat#D3072
EDTA	Sigma-Aldrich	Cat# E9884
FeSO_4_·7H_2_O	Sigma-Aldrich	Cat#215422
Hydrochloric acid	Sigma-Aldrich	Cat#1101652500
Hydrogen peroxide	Sigma-Aldrich	Cat#1086001000
MnCl_2_·4H_2_O	Sigma-Aldrich	Cat#M3634
NaCl	Sigma-Aldrich	Cat#S9888
NaOH	Sigma-Aldrich	Cat#S5881
Na_2_MoO_4_·2H_2_O	Sigma-Aldrich	Cat#M1003
Sodium dodecyl sulfate	Sigma-Aldrich	Cat#L3771
Uridine	Merck	Cat#U3750
ZnSO_4_·7H_2_O	Sigma-Aldrich	Cat#Z0251

**Software and algorithms**		

ImageJ/Fiji	(Schindelin et al.^4^)	https://imagej.net/software/fiji/downloads
Morphology of Pelleted and Dispersed Mycelia plugin	Supplemental information	Not applicable

**Other**		

Miracloth	Sigma-Aldrich	Cat#475855
Zeiss Axioscope 5	Zeiss Microscopy	Not applicable
Leica DM5000	Leica Microsystems	Not applicable
Olympus SZX7	Olympus Microsystems	Not applicable
Canon DS126251	Canon	Not applicable
Heratherm General Protocol Incubator	Thermo	Cat#51028130
INFORS HT Multitron Standard	INFORS HT	Not applicable

## Materials and equipment

### Trace element solution

In a beaker, prepare ∼500 mL double-distilled water. Add the compounds in the order they are presented in the recipe table, adjusting the pH to 6.0 using 1 M NaOH after addition of each compound. When all components are dissolved, adjust the final volume to 1 L using double-distilled water and adjust the pH to 4.0 using 1 M HCl. Finally, filter sterilize.

**Table:** 

Reagent	Final concentration	Amount
EDTA	1.00%	10.00 g
ZnSO_4_·7H_2_O	0.44%	4.40 g
MnCl_2_·4H_2_O	0.10%	1.01 g
CoCl_2_·6H_2_O	0.032%	0.32 g
CuSO_4_·5H_2_O	0.032%	0.32 g
Na_2_MoO_4_·H_2_O	0.03%	0.30 g
CaCl_2_	0.11%	1.47 g
FeSO_4_·7H_2_O	0.10%	1.00 g
ddH_2_O	N/A	to 1 L

Store at room temperature for up to one month.

### Asp + N 50×

Adjust the final concentration to 500 mL when all components are dissolved and autoclave at 121°C for 20 min.

**Table:** 

Reagent	Final concentration	Amount
KCl	350 mM	13.04 g
KH_2_PO_4_	550 mM	37.42 g
NaN0_3_	3.5 M	148.73 g
ddH_2_O	N/A	to 500 mL

Store at room temperature for up to one month.

### MgSO_4_ 500×

Autoclave at 121°C for 20 min once dissolved.

**Table:** 

Reagent	Final concentration	Amount
MgS0_4_	1 M	120.36 g
ddH_2_O	N/A	to 1 L

Store at room temperature for up to one month.

### Glucose 50×

Prepare in warm (50°C–60°C water). When glucose is fully dissolved, make up to 1 L and autoclave at 121°C for 20 min.

**Table:** 

Reagent	Final concentration	Amount
Glucose	50% (w/v)	500 g
ddH_2_O	N/A	to 1 L

Store at room temperature for up to one month.

### Minimal media (MM)

• Calculate the amount of water necessary for the final volume of MM (see table below).
• For solid media, autoclave water at 121°C for 20 min with 1.5% (w/v) agar.
• Allow to cool to 50°C in a water bath or temperature-controlled heating oven before adding the following:

**Table:** 

Reagent	Final concentration	Amount
Asp + N	1 ×	5 mL
Glucose 50 ×	1% (v/v)	5 mL
MgSO_4_	2 mM	500 μL
Trace element solution	1 ×	250 μL
ddH_2_O	N/A	to 250 mL

Minimal media should be kept molten at 50°C–65°C and poured as agar plates the same day. Minimal media plates can be stored at 4°C for up to a week before use. MM plates supplemented with doxycycline should be stored at 4°C in the dark for a maximum of 24 h before use.

**CRITICAL:** For auxotrophic *A. niger* mutants, add necessary supplements after autoclaving. For example, for uridine auxotrophs, add filter sterilized uridine to a final concentration of 4 mM.

***Optional:*** For titration of gene expression using the tet-on cassette, supplement MM with 0, 0.2, 2, or 20 μg/mL doxycycline immediately prior to pouring the plate.

### Microscope and camera

For counting fungal spores, a standard light microscope and counting chamber are required. We use an Ernst Leitz Wetzlar microscope and a 25 × objective, with a Paul Marienfeld Neubauer Improved counting chamber (catalog number 0640810).

For MPD analysis of fungal macromorphology, a range of microscopes and camera setups are appropriate; three examples we have used are:

• Zeiss Axioscope 5.
• Leica DM5000.
• Olympus SZX7 stereomicroscope connected to a Canon DS126251.

### Static incubator

This protocol requires a general laboratory static incubator. We use a Thermo Scientific Heratherm General Protocol Incubator.

### Shaking incubator

This protocol requires a general laboratory shaking incubator. We use an INFORS HT Multitron Standard.

## Step-by-step method details

### Prepare individual spore solutions of progenitor and mutant isolates

**Timing: 1 day preparation, 10 days incubation**

Purified *A. niger* spores suspended in water with a defined density are required for inoculating solid and liquid growth media.

1. Inoculate solid growth media from long term storage.
  a. Pour minimal media (MM) plates and air dry (1 plate per strain). See materials and equipment section for MM recipe.
  b. Retrieve strains from long term storage. We store isolates as spore suspensions in 25% glycerol stocks at −80°C.
    i. Keep glycerol stocks on ice and do not allow to defrost.
    ii. Gently touch frozen spore solution with a sterile cotton stick and streak to a single colony forming unit (CFU).
  c. Incubate plates at 30°C in a static, temperature-controlled incubator for 3–5 days until single CFUs are visible.
2. Subculture single colony.
  a. Pour MM plates and air dry (5 plates per strain).
  b. Using a sterile cotton stick, isolate a single CFU and subculture in a grid pattern (Figure 1).

**Figure 1:**
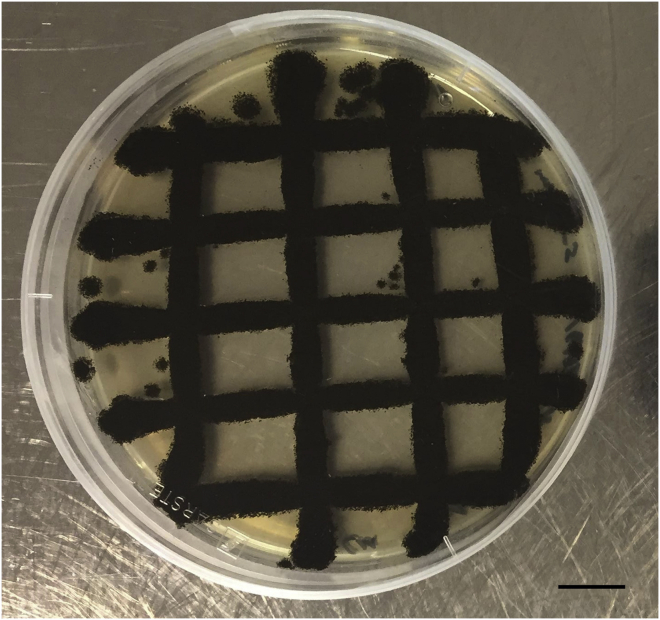
Exemplar image of *A. niger* growing on agar plate as grid format Sub-culturing as a grid allows visual inspection of plates for fungal contamination, e.g., by colonies growing at a different rate, sporulation phenotype, or spore color. Scale bar is 1 cm.
  c. Incubate plates at 30°C for 5 days or until abundantly sporulated.
3. Harvest spores.
  a. Prior to harvesting spores, inspect the plate for contamination.
  b. Add approximately 10 mL sterilized water to the agar plate, and gently agitate the surface with a sterile cotton stick to release spores.
  c. Remove agar debris by filtering spore solution through two layers of sterile Miracloth.
  d. Add sterilized water to a final volume of 30 mL, and vortex for 1 min.
  e. Centrifuge for 10 min at 2750 RCF.
  f. Remove supernatant, and repeat wash step one more time (d. and e.). Suspend spores in 5 mls sterile water.
  g. Enumerate spores using a counting chamber, and adjust to a final density of 1 × 10^5^/mL (for solid growth assays) and 2 × 10^8^/mL (liquid growth assays). Purified spores can be stored at 4°C and used within two weeks.

**CRITICAL:** To avoid cross contamination of genetically distinct *A. niger* strains, thoroughly decontaminate all working areas and equipment between spore preparation of each isolate. We routinely do this by decontaminating work surfaces using 2% Kohrsolin® FF and 15-minute periods of UV irradiation.

**CRITICAL:** For preparation of sterile Miracloth filters, cut two 10 × 10 cm squares and store both in aluminum foil. Autoclave at 121°C for 20 min and store at room temperature prior to use.

### Determine conditions that cause sublethal growth perturbation in progenitor control on solid agar

**Timing: 1 day preparation, 5 days incubation**

Sublethal growth perturbation in the progenitor strain on solid agar must first be established as (i) insufficient stress will not cause a measurable reduction in colony radial growth, and (ii) excess stress may lead to total inhibition of cell growth or death. For each progenitor strain it is therefore necessary to titrate desired stress conditions in preliminary growth assays. We typically define sublethal perturbation as a 5%–50% reduction in radial growth rates under stress conditions when compared to standard growth media controls. Here, we use hydrogen peroxide enacting oxidative stress as an exemplar. This assay should be conducted in technical triplicate.

4. Prepare agar plates.
  a. Using a permanent pen, mark the center spot on the back of a petri dish. Draw two lines at 180° angles through the center mark.
  b. Pour minimal media agar supplemented with 0, 0.5, 1, 10 or 100 mM H_2_O_2_.

**CRITICAL:** Check stability of stress compounds during autoclaving. If necessary, add filter sterilized solutions after agar has cooled sufficiently.

**CRITICAL:** Plates must be fully dried. Droplets of water on the agar surface will result in defective spore spotting.

***Optional:*** Conduct a preliminary literature analysis to estimate concentrations/conditions that will result in sublethal stress prior to pouring plates.

5. Inoculate plates.
  a. Vortex the 1 × 10^5^ spore/mL solution of progenitor strain for a minimum of 1 min.
  b. Carefully add 10 μL of this solution to the marked center of each agar plate.
  c. Allow inoculum to fully dry on agar surface.
6. Incubate for 5 days at 30°C.
7. Measure radial growth rates.
  a. Use a vernier caliper and measure from the center of the colony to the outermost hyphal edge. We conduct this on the back on the agar plate and record to the nearest 0.5 mm.
  b. Measure radial growth at the four axes indicated by the marked lines (see step 4a).

**CRITICAL:** Radial growth rates should be measured visually. Automated image analysis can result in inaccurate calls of the extending hyphal front.

8. Calculate reduced growth rates thus:

$$\left(\frac{\text{Average}\;\text{radial}\;\text{growth}\;\text{rate}\;\left(\text{mm}\right)\;\text{on}\;\text{MM}+\text{stress}}{\text{Average}\;\text{radial}\;\text{growth}\;\text{rate}\;\left(\text{mm}\right)\;\text{on}\;\text{MM}}\right)\times 100$$

A 5%–50% reduction in radial growth on MM + stress relative to MM is appropriate for subsequent steps of this protocol.

### Screen mutant and progenitor isolates on sublethal stress and control agar

**Timing: 1 day preparation, 5 days incubation**

Analysis on radial growth rates on solid agar (+/- stress) enables the calculation of susceptibility coefficients. This assay should be conducted in technical triplicate. Sublethal perturbation that we routinely use for isolate MA70.15 are given in Table 1.

9. Prepare agar plates as follows:
  a. See step 4. for marking the center and measurement axes of the agar plate.
  b. Per strain/technical replicate, generate four MM plates supplemented with 0, 0.2, 2 or 20 μg/mL doxycycline.
  c. Per strain/technical replicate, generate MM plus stress (e.g., 1 mM H_2_O_2_) supplemented with 0, 0.2, 2 or 20 μg/mL doxycycline.
  d. Allow plates to fully dry to avoid moisture on the surface.

**CRITICAL:** Doxycycline is light sensitive, and therefore light exposure should be minimal. We therefore avoid unnecessary light exposure, e.g., by turning off room lights while plates dry. Additionally, we wrap doxycycline supplemented plates with tin foil during storage or incubation.

***Optional:*** Where using strains that are not tet-on conditional expression mutants (e.g., conventional deletion/overexpression or mutagenized isolates) it is not necessary to add doxycycline to growth media.

10. Inoculate plates, incubate, and measure radial growth as detailed in steps 5, 6 and 7.

***Optional:*** We routinely measure radial growth rates at days 3, 5 and 10. If desired, a single timepoint can be used.

11. Record colony images.
  a. Inspect the lids of agar plates for condensation and if necessary either air dry in a category II microbiological hood, or wipe dry with sterile paper towels.
  b. Remove permanent marker on rear of plate using 95% ethanol or acetone.
  c. Mount a camera on a tripod. We typically use mobile phone cameras, e.g., iPhone or similar.
  d. Place agar plates on a light background and capture an image of the colony.

***Optional:*** Images of the back side of the colony can also be captured, which can be informative for noting different pigmentation between mutant and control strains.

***Optional:*** We routinely capture colony images at days 3, 5 and 10. To reduce work load and data storage, a single timepoint can be used.

**Table 1:** Exemplar conditions for sublethal stress in *A. niger* MA70.15 and derivative mutants

Process or subcellular location	Stressor or growth condition	Final concentration
Oxidative stress response	H_2_O_2_	1 mM
Heat shock stress response	42°C	–
Osmotic stress	NaCl	0.2 M
Protein folding	SDS	0.005%
pH adaptation (low)	pH 4	–
pH adaptation (high)	pH 7.5	–
Low carbon	Glucose	0.1%
Cell wall (glucan synthesis)	Caspofungin diacetate	1 μg/mL
Cell wall (chitin perturbation)	Congo red	200 μg/mL

### Quantification of mutant and progenitor control in submerged culture using MPD analysis

**Timing: 0.5 day preparation, 3 days incubation**

MPD analysis provides multiple measurements of submerged macromorphologies, including a quantification of culture heterogeneity (% pelleted growth), structure diameter, aspect ratio, solidity, area, and morphology number. This assay should be conducted in technical triplicate.

12. Inoculate liquid growth cultures.
  a. Prepare 100 mL Erlenmeyer flasks containing 20 mL MM supplemented with 0, 0.2, 2 or 20 μg/mL doxycycline.
  b. Inoculate media with 100 μL of 2 × 10^8^ spore suspension from progenitor control or mutant isolate(s). Final density is 1 × 10^6^ spores/mL.
  c. Incubate flasks at 30°C, 220 RPM in the dark for 72 h.

**CRITICAL:** Trace amounts of detergent in sterilized glass wear can drastically impact morphological development of *A. niger*. Thoroughly wash all glass wear with distilled water prior to sterilization.

**CRITICAL:** Confirm all flasks are of identical design, as deviations (e.g., in circumference of flask neck) can modify fungal macromorphology.

**CRITICAL:** Flasks are sealed using sterilized cotton plugs or similar. Note that plugs or other method of sealing should be uniform between each flask.

13. Capture images of fungal culture.
  a. Decant culture and biomass into a clean 50 mL falcon tube and invert gently several times to separate biomass.
  b. Pour 5–10 mls into a 25 mL petri dish.
  c. Capture images of fungal culture using a desired magnification. We routinely use ∼10–20 × magnification which captures large pellets (e.g., 3 mm diameter) and dispersed mycelial fragments.
  d. If you observe pelleted macromorphologies, ensure that >10 pellets are captured per fungal culture. Multiple images are advised.

**CRITICAL:** In order to define the μm/pixel ratio for quantification, capture an image of a known length (e.g., a technical ruler) at the identical magnification used for analyzing fungal macromorphology.

***Optional:*** Where macromorphological fungal structures and physically touching, gently shake the petri dish to separate them. If required, use a sterile pipette tip to physically separate them. In rare instances where biomass is very high, we dilute the culture media with either MM or distilled water immediately prior to imaging in order to separate structures. We have found that a 1:1 dilution is sufficient to separate macromorphologies for imaging.

## Expected outcomes

### Growth on solid agar

**Figure 2:**
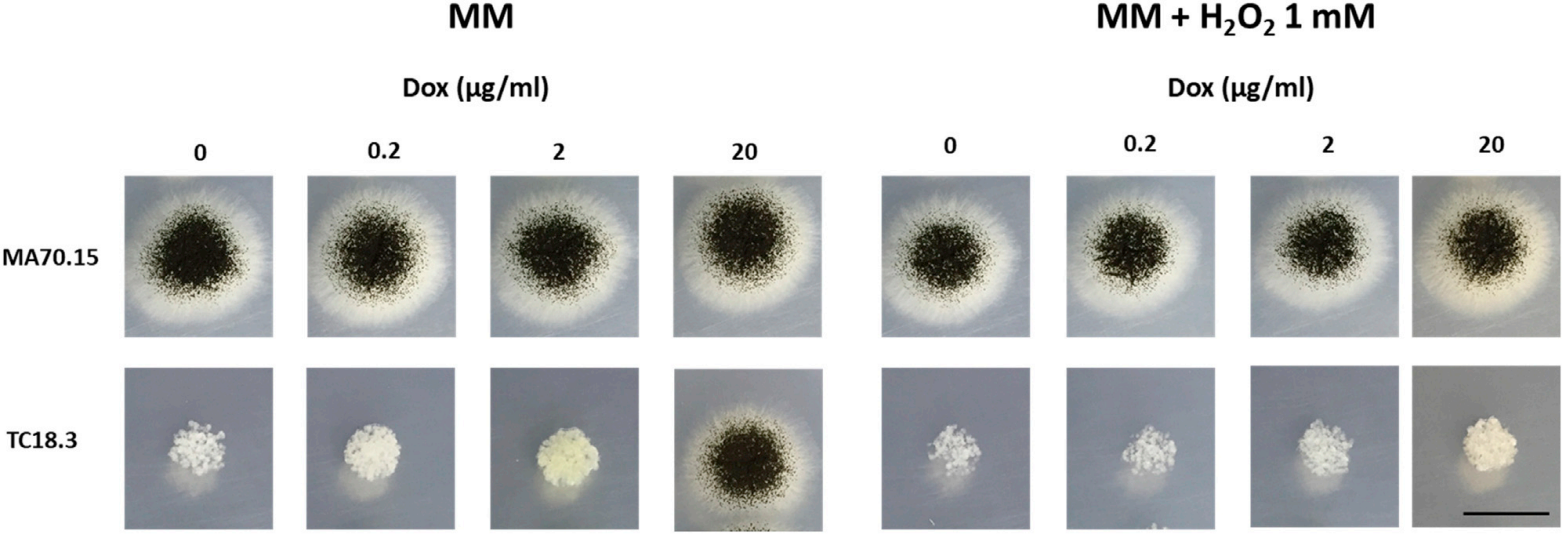
Colony growth on control and stress conditions for mutant TC18.3 and progenitor isolate MA70.15 Titration of gene expression using doxycycline (Dox) concentrations are indicated. Note minor reduction of MA70.15 radial growth and sporulation on MM + H_2_O_2_ when compared to MM control. Note marked reduction in radial growth and conidiation for strain TC18.3 when grown on H_2_O_2_ and 20 μg/mL doxycycline. Scale bar is 1 cm.

This protocol will generate a set of average radial growth rates for mutant and progenitor strains on control and stress growth media. These data are visualized by images of colony growth, an exemplar of which is show for tet-on conditional expression mutant TC18.3 and progenitor control MA70.15 under hydrogen peroxide stress (Figure 2).

### Growth in liquid culture

**Figure 3:**
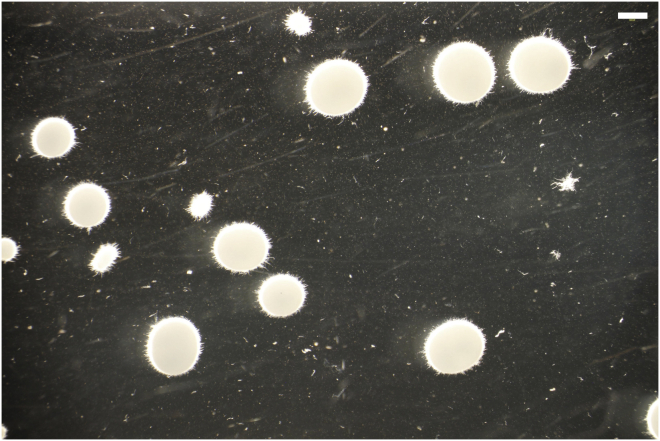
Exemplar image of isolate MA70.15 in submerged culture Culture conditions were MM, 0 μg/mL Dox, 30°C, 220 RPM, 72 h in the dark. Scale bar in top right of image is 1 cm.

This protocol will produce a compendium of culture images (Figure 3). These images constitute the raw data for MPD quantification.

## Quantification and statistical analysis

### Growth assay on solid agar

**Figure 4:**
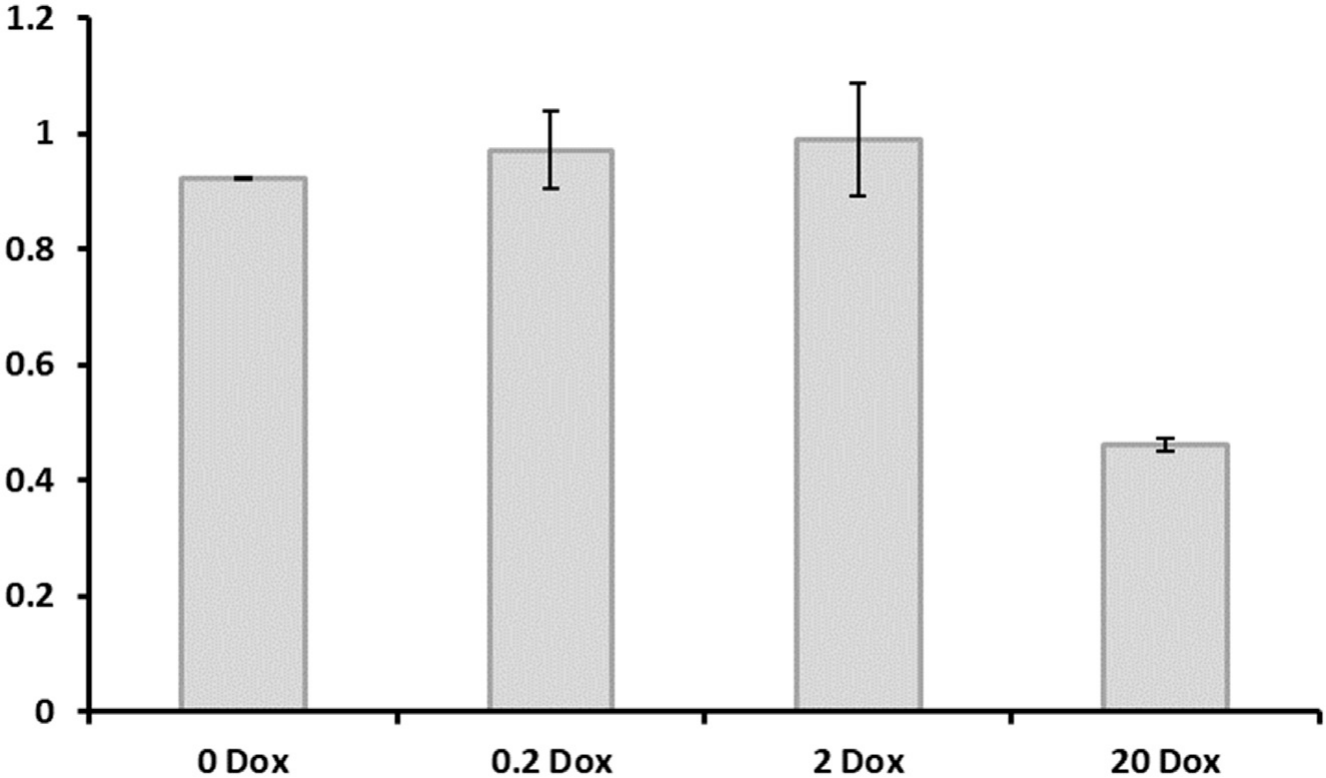
Susceptibility coefficients for isolate TC18.3 when grown on 1 mM H_2_O_2_ Exemplar data are derived from the assay depicted in Figure 2. Note this strain is highly susceptible to H_2_O_2_ stress when the gene is expressed at high levels (20 μg/mL Dox). Error bars are from triplicate replicates. Based on these data, we hypothesize that the gene expressed under the tet-on cassette in strain TC18.3 (An01g02600, *aplD*) impacts cellular response to sublethal H_2_O_2_ challenge when over-expressed. By increasing this assay for multiple stress conditions, more detailed hypotheses regarding gene function can be postulated.

1. Calculate and visualize susceptibility coefficients.
  a. *A. niger* susceptibility coefficients for each technical replicate are calculated thus:
    $$\frac{\text{Radial}\;\text{growth}\;\text{rate}\;\text{mutant}\;\left(\text{MM}\right)}{\text{Radial}\;\text{growth}\;\text{progenitor}\;\left(\text{MM}\right)}/\frac{\text{Radial}\;\text{growth}\;\text{rate}\;\text{mutant}\;\left(\text{MM}+\text{stress}\right)}{\text{Radial}\;\text{growth}\;\text{rate}\;\text{progenitor}\;\left(\text{MM}+\text{stress}\right)}$$
  b. Visualize coefficients using bar graphs and report standard deviation across technical replicates (Figure 4).

### MPD analysis of liquid culture

2. Organize images and define analysis parameters.
  a. Generate a directory of all culture images to be analyzed. Multiple images taken of a single culture (Figure 3) should be organized into a single folder.
  b. Open MPD (choose: plugins > MPD).
  c. Choose the input directory containing files to be quantified.
  d. Create an output directory where analyzed data is deposited.
  e. Confirm if images are light on dark background or vice versa.
  f. Define the resolution (μm/pixel) ratio. We routinely do this using the ‘straight line’ selection tool in Image J and by capturing an image of a known length (see Critical Point, step 13). Click on one end of the known length, and drag a straight line to the other end of the known length. Next, select, ‘Analyze’ and then ‘Set Scale’, which will give the precise distance in of the known length in pixels. Calculate the resolution (μm/pixel) ratio by dividing the number of μm of the known length by the measured number of pixels.
  g. Define thresholds for distinguishing dispersed/pellet macromorphologies (based on structure area, μm^2^).
  h. Define a minimal cut-off size to remove artefacts (based on structure area, μm^2^).
  i. Run MPD analysis.

***Optional:*** Users can select default parameters for points g. (dispersed mycelia are defined as having and area < 500 μm^2^ and ≥ 95 μm^2^, and pellets have an area > 500 μm^2^) and h. (all objects with an area < 95 μm^2^ are considered artefacts).

**Figure 5:**
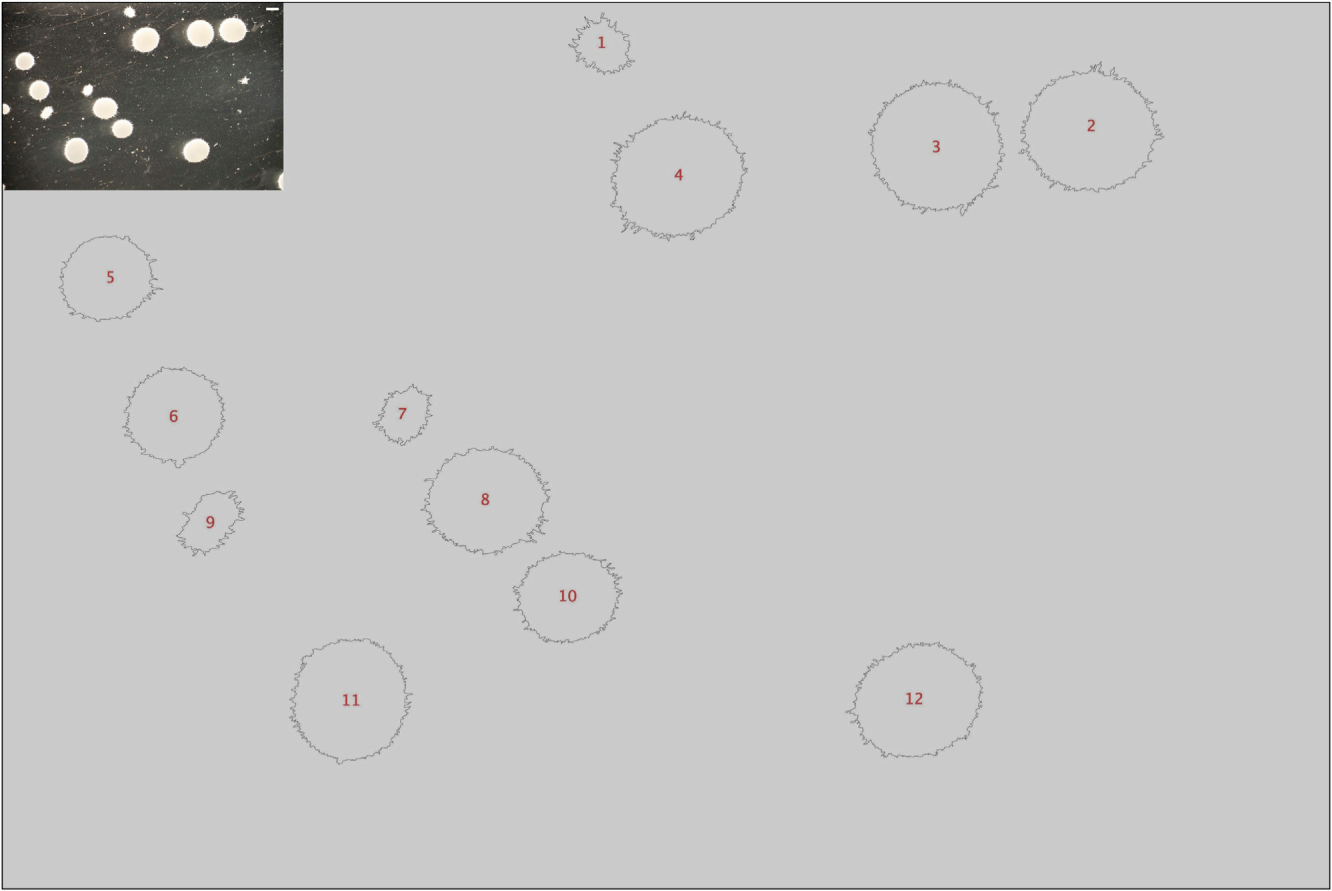
Exemplar quality control image from updated MPD plugin Comparison between original (top left) and quality control image enable visual inspection for the accuracy of automated pellet macromorphological calls. Shown is pelleted output file (additional dispersed output file not shown). Note that red index numbers in the center of each pellet enable a user to omit poor calls from downstream analysis.
  b. We routinely display MPD output for pelleted growth forms (diameter, area, aspect ratio, solidity, morphology number) as box whisker plots depicting average, median, upper/lower quartiles, and outliers.
  c. We routinely visualize data using boxplots (Figure 6).

3. Quality control and data processing/visualization.
  a. Check accuracy of automated analysis using the generated quality control images (Figure 5). Poor calls (e.g., where the pellet boundaries are missed) can be omitted from downstream analysis based on their index number.

**Figure 6:**
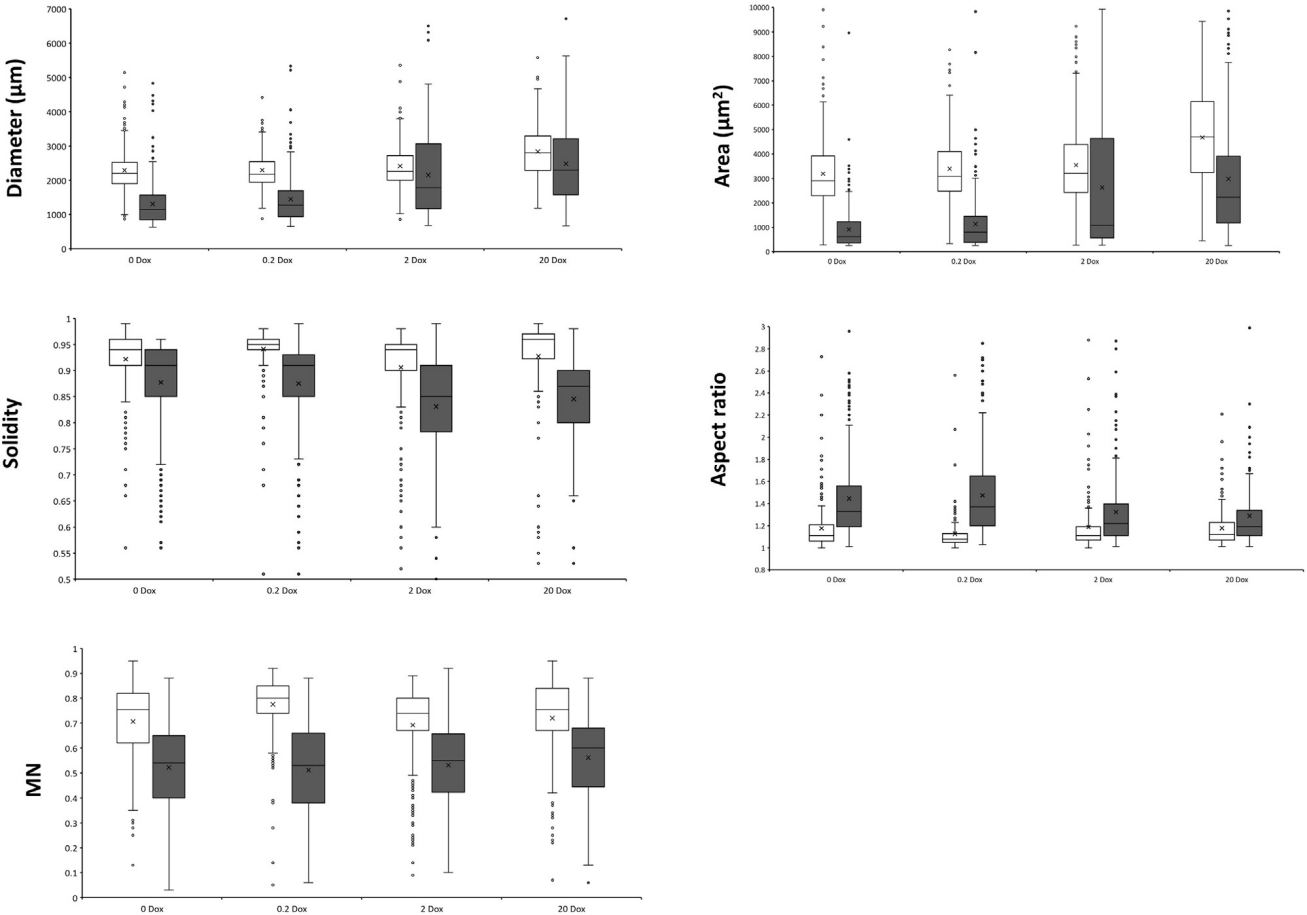
Exemplar box whisker plots comparing pellet macromorphology of strain TC18.1 with MA70.15 control in submerged culture using MPD MA70.15 and TC18.1 are depicted using unshaded and shaded boxplots, respectively. Crosses depict mean values. Outliers are given as circles.

### Data interpretation

In order to aid interpretation of these assays we routinely visualize data in matrix format (Table 2).

**Table 2:** Titration of *aplD* expression drastically impacts *A. niger* growth on solid and in liquid culture

Strain	Dox	Solid			Pelleted growth in liquid culture					
		H_2_O_2_	pH	Caspo	Diameter	Area	Solidity	Aspect ratio	MN	% Pellet
TC18.1	0	–	–	<<<	<	<<<	<	>>>	<<	<
	0.2	–	–	<<<	<	<<<	<	>>>	<<	<
	2	–	–	<<	–	<	<	>>>	<<	–
	20	<<<	–	–	–	–	<	>>>	<<	–

No change relative to MA70.15 control is represented by a dash. Increase and decrease are represented by >, <, respectively. Note, magnitude of change relative to control is represented by the number of arrows, e.g., low, moderate, and high by one, two, or three arrows, respectively.

Based on these data, the above hypotheses regarding gene *aplD* can be postulated in *A. niger*: (i) elevated expression inhibits cellular response(s) to hydrogen peroxide-based oxidative stress; (ii) high expression is necessary for successful response to caspofungin, (iii) *aplD* expression has multiple and diverse impacts on macromorphological formation during submerged culture; (iv) poor response to cell wall perturbation (i.e., caspofungin) may be correlated with reduced pellet diameter, area, and pellet formation. Note, that when multiple strains are incorporated into such data matrix, correlations e.g., between cell wall perturbation and pellet diameter, become more robust and easier to delineate.^2^

## Limitations

Users of this protocol should be aware of several limitations. Firstly, measurements of radial growth are somewhat limited in that they cannot measure all defects in fungal development. For example, a mutant may be unable to sporulate under specific stress conditions, yet the radial growth rate could be unchanged relative to controls.

Additionally, the susceptibility coefficient method is limited for study of essential genes, whereby omission of dox to growth media results in zero growth. We routinely record these susceptibility coefficients at 0 μg/mL dox as *1*, given that there is no change in growth between mutant and control strains on MM or MM + stress conditions (i.e., both do not grow). However, this approach may not be entirely appropriate, especially where titration of the essential gene using 0.2/2/20 μg/mL dox results in high sensitivity of an essential mutant relative to control. This can result in an artefact, where 0 μg/mL dox reports a value of *1* and 0.2/2/20 μg/mL can result in susceptibility coefficients <1. In this instance, it may be more appropriate to report the susceptibility coefficient as ‘not applicable’ or similar*.*

## Troubleshooting

### Problem 1

High inter-replicate variation in strain macromorphology during shake flask culture, protocol steps 12 and 13.

### Potential solution

• Thoroughly remove traces of detergent from all glass ware prior to autoclaving.
• Check density of spore inoculum using serial dilution. Poor reproducibility can be due to low spore counts (< 1 × 10^6^ spores per mL final concentration).
• Confirm incubator conditions (temperature, RPM) are stable. Minor variations (e.g., in temperature, light) can drastically affect macromorphological formation.
• Confirm stability of the tet-on cassette at the target locus when grown in media lacking selection. We routinely do this using PCR analyses from genomic DNA extracted from culture biomass. following 72 h cultivation (see Zheng et al.^5^) for details on confirming homokaryon strains using PCR).

## Resource availability

### Lead contact

Further information and requests for resources and reagents should be directed to and will be fulfilled by the lead contact, Prof Vera Meyer, vera.meyer@tu-berlin.de.

### Materials availability

This study did not generate new unique reagents.

## Data Availability

Updated MPD analysis plugin is available as Data S1. Datasets generated in this study are available from the lead contact upon request.

## Supplemental information

Data S1. MPD plugin, related to section "quantification and statistical analysis," step 2

## Footnotes

Supplemental information can be found online at https://doi.org/10.1016/j.xpro.2022.101883.
